# [2-(Benzyl­idene­amino)-4,5,6,7-tetra­hydro­benzo[*b*]thio­phen-3-yl](phen­yl)methanone

**DOI:** 10.1107/S1600536814006679

**Published:** 2014-04-02

**Authors:** Manpreet Kaur, Jerry P. Jasinski, Channappa N. Kavitha, Hemmige S. Yathirajan, K. Byrappa

**Affiliations:** a Department of Studies in Chemistry, University of Mysore, Manasagangotri, Mysore 570 006, India; b Department of Chemistry, Keene State College, 229 Main Street, Keene, NH 03435-2001, USA; c Materials Science Center, University of Mysore, Vijyana Bhavan Building, Manasagangothri, Mysore 570 006, India

## Abstract

In the title compound, C_22_H_19_NOS, the cyclo­hexene ring of the tetra­hydro­benzo­thio­phenyl ring system adopts a slightly distorted half-chair conformation and is twisted slightly [7.5 (8)° for the major disorder component] from the mean plane of the thio­phene ring. The dihedral angles between the mean planes of the thio­phene ring and the phenyl rings are 65.7 (3) and 8.3 (4)°. The phenyl rings are twisted with respect to each other by 73.8 (7)°. Disorder was modeled for four C atoms of the cyclo­hexene ring over two sets of sites with an occupancy ratio of 0.659 (2):0.341 (2). In the crystal, a single weak C—H⋯O inter­action links the mol­ecules into [001] chains.

## Related literature   

For the importance of thio­phene derivatives, see: Molvi *et al.* (2007 ▶); Rai *et al.* (2008 ▶); Asthalatha *et al.* (2007 ▶). For applications of 2-amino­thio­phene derivatives, see: Sabnis *et al.* (1999 ▶); Puterová *et al.* (2010 ▶); Cannito *et al.* (1990 ▶); Nikolakopoulos *et al.* (2006 ▶); Lütjens *et al.* (2005 ▶). For the biological and industrial importance of Schiff bases, see: Desai *et al.* (2001 ▶); Karia & Parsania (1999 ▶); Samadhiya & Halve (2001 ▶); Singh & Dash (1988 ▶); Aydogan *et al.* (2001 ▶); Taggi *et al.* (2002 ▶). For a related structure, see: Kubicki *et al.* (2012 ▶). For puckering parameters, see Cremer & Pople (1975 ▶). For standard bond lengths, see: Allen *et al.* (1987 ▶).
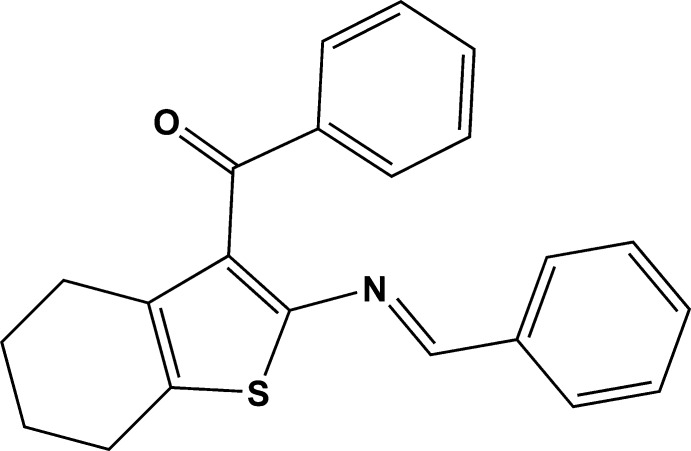


## Experimental   

### 

#### Crystal data  

C_22_H_19_NOS
*M* *_r_* = 345.44
Monoclinic, 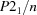

*a* = 8.78760 (16) Å
*b* = 14.0091 (3) Å
*c* = 14.4120 (2) Å
β = 94.8913 (17)°
*V* = 1767.75 (5) Å^3^
*Z* = 4
Mo *K*α radiation
μ = 0.19 mm^−1^
*T* = 173 K
0.24 × 0.22 × 0.12 mm

#### Data collection  

Agilent Eos, Gemini diffractometer
Absorption correction: multi-scan (*CrysAlis PRO*; Agilent, 2012 ▶) *T*_min_ = 0.896, *T*_max_ = 1.000
22680 measured reflections
6047 independent reflections
4745 reflections with *I* > 2σ(*I*)
*R*_int_ = 0.029

#### Refinement  

*R*[*F* ^2^ > 2σ(*F* ^2^)] = 0.043
*wR*(*F* ^2^) = 0.113
*S* = 1.03
6047 reflections
239 parameters
48 restraints
H-atom parameters constrained
Δρ_max_ = 0.39 e Å^−3^
Δρ_min_ = −0.25 e Å^−3^

### 

Data collection: *CrysAlis PRO* (Agilent, 2012 ▶); cell refinement: *CrysAlis PRO*; data reduction: *CrysAlis PRO*; program(s) used to solve structure: *SUPERFLIP* (Palatinus & Chapuis, 2007 ▶); program(s) used to refine structure: *SHELXL2012* (Sheldrick, 2008 ▶); molecular graphics: *OLEX2* (Dolomanov *et al.*, 2009 ▶); software used to prepare material for publication: *OLEX2*.

## Supplementary Material

Crystal structure: contains datablock(s) I. DOI: 10.1107/S1600536814006679/zs2292sup1.cif

Structure factors: contains datablock(s) I. DOI: 10.1107/S1600536814006679/zs2292Isup2.hkl

Supporting information file. DOI: 10.1107/S1600536814006679/zs2292Isup3.cml

CCDC reference: 993683

Additional supporting information:  crystallographic information; 3D view; checkCIF report

## Figures and Tables

**Table 1:** Hydrogen-bond geometry (Å, °)

*D*—H⋯*A*	*D*—H	H⋯*A*	*D*⋯*A*	*D*—H⋯*A*
C18—H18⋯O1^i^	0.95	2.45	3.4034 (15)	176

Symmetry code: (i) 
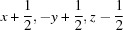
.

**Fig. 1.**
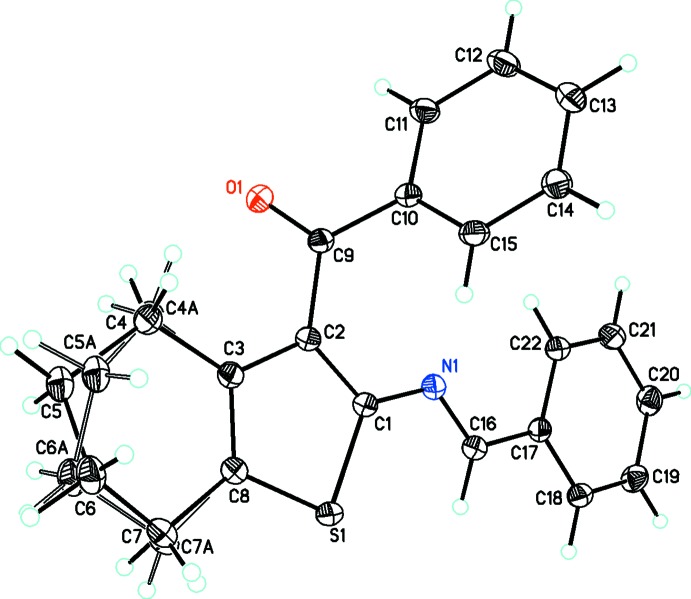
An ORTEP drawing of (I) showing the labeling scheme of the molecule with 30% probability displacement ellipsoids. Disorder is shown modeled for the C4, C5, C6 and C7 carbon atoms of the tetrahydrobenzothiophenyl ring over two sites with an occupancy ratio of 0.659 (2):0.341 (2).

**Fig. 2.**
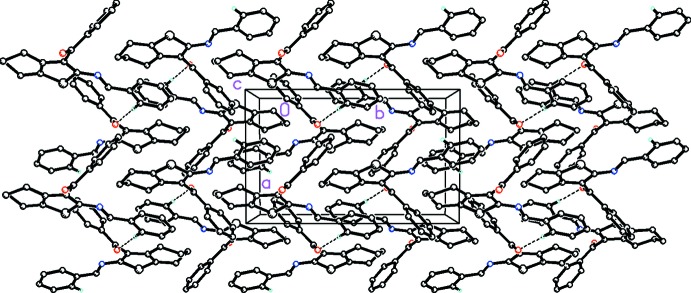
Molecular packing for (I) viewed along the c axis. Dashed lines indicate weak C—H···O intermolecular interactions which link the molecules into dimers. H atoms not involved in hydrogen bonding have been removed for clarity.

## supplementary crystallographic information

### 1. Comment

Thiophene derivatives have been reported to exhibit a broad spectrum of biological properties such as anti-inflammatory, analgesic, antidepressant, antimicrobial and anticonvulsant activities (Molvi *et al.*, 2007; Rai *et al.*, 2008; Asthalatha *et al.* , 2007). 2-Aminothiophene derivatives have been used in a number of applications in pesticides, dyes and pharmaceuticals. A review on the synthesis and properties of these compounds was reported in 1999 by Sabnis *et al.* and more recently by Puterová *et al.* (2010). Substituted 2-aminothiophenes are active as allosteric enhancers at the human A1 adenosine receptor (Cannito *et al.*, 1990; Nikolakopoulos *et al.*, 2006; Lütjens *et al.*, 2005). Schiff base compounds are an important class of compounds both synthetically and biologically. These compounds show biological properties including antibacterial, antifungal, anticancer and herbicidal activities (Desai *et al.*, 2001; Karia & Parsania, 1999; Samadhiya & Halve, 2001; Singh & Dash, 1988). Furthermore, Schiff bases are utilized as starting materials in the synthesis of compounds of industrial (Aydogan *et al.*, 2001) and biological interest such as b-lactams (Taggi *et al.*, 2002). The crystal structures and molecular structures of two 2-aminothiphenes have been previously reported by our group (Kubicki *et al.*, 2012). In view of the importance of 2-aminothiphenes and Schiff bases, we report the crystal structure of the Schiff base of the previously reported 2-aminothiophene, C_22_H_19_NOS, (I).

The title compound (I) crystallizes with one independent molecule in the asymmetric unit (Fig. 1). In the molecule, the cyclohexene moiety of tetrahydrobenzothiophenyl ring adopts a slightly distorted half-chair conformation and is slightly twisted from the mean plane of the thiophene ring by 7.5 (8)° for the major disorder component. The dihedral angle between the mean planes of the thiophene ring and the two phenyl rings is 65.7 (3)° (C10–C15) and 8.3 (4)° (C17–C22), respectively. The two phenyl rings are twisted with respect to each other by 73.8 (7)°. Disorder was modeled for the C4, C5, C6 and C7 carbon atoms of the cyclohexene ring over two sites with occupancy ratios of 0.659 (2):0.341 (2). Bond lengths are within normal ranges (Allen *et al.*, 1987). A single weak intermolecular C18—H···O1^i^ hydrogen-bonding interaction (Table 1) links the molecules into dimers (Fig. 2) but no classical hydrogen bonds are present.

### 2. Experimental

To a solution of (2-amino-4,5,6,7-tetrahydrobenzo[b]thiophen-3-yl)- phenylmethanone (200 mg, 0.79 mmol) in 10 ml of methanol, an equimolar amount of benzaldehyde (84 mg, 0.79 mmol) was added dropwise with constant stirring. The mixture was refluxed for 4 hours. A yellow precipitate was obtained. The reaction completion was confirmed by thin layer chromatography. The precipitate was filtered and dried at room temperature overnight. The solid was recrystallized from dichloromethane and the crystals were used as such for the X-ray diffraction studies.

### 3. Refinement

All of the H atoms were placed in their calculated positions and then refined using the riding model with C—H bond lengths of 0.95 Å (CH) or 0.99 Å (CH_2_). Isotropic displacement parameters for these atoms were set to 1.2 (CH, CH_2_) times *U*_eq_ of the parent atom. Disorder was modeled for the C4, C5, C6 and C7 carbon atoms of the tetrahydrobenzothiophenyl ring over two sites with an occupancy ratio of 0.659 (2):0.341 (2).

### Crystal data

**Table:**

C_22_H_19_NOS	*F*(000) = 728
*M**_r_* = 345.44	*D*_x_ = 1.298 Mg m^−^^3^
Monoclinic, *P*2_1_/*n*	Mo *K*α radiation, λ = 0.71073 Å
*a* = 8.78760 (16) Å	Cell parameters from 6837 reflections
*b* = 14.0091 (3) Å	θ = 3.9–32.5°
*c* = 14.4120 (2) Å	µ = 0.19 mm^−^^1^
β = 94.8913 (17)°	*T* = 173 K
*V* = 1767.75 (5) Å^3^	Irregular, yellow
*Z* = 4	0.24 × 0.22 × 0.12 mm

### Data collection

**Table:**

Agilent Eos, Gemini diffractometer	6047 independent reflections
Radiation source: Enhance (Mo) X-ray Source	4745 reflections with *I* > 2σ(*I*)
Detector resolution: 16.0416 pixels mm^-1^	*R*_int_ = 0.029
ω scans	θ_max_ = 32.9°, θ_min_ = 3.0°
Absorption correction: multi-scan (*CrysAlis PRO*; Agilent, 2012)	*h* = −13→13
*T*_min_ = 0.896, *T*_max_ = 1.000	*k* = −20→18
22680 measured reflections	*l* = −21→21

### Refinement

**Table:**

Refinement on *F*^2^	Primary atom site location: structure-invariant direct methods
Least-squares matrix: full	Hydrogen site location: inferred from neighbouring sites
*R*[*F*^2^ > 2σ(*F*^2^)] = 0.043	H-atom parameters constrained
*wR*(*F*^2^) = 0.113	*w* = 1/[σ^2^(*F*_o_^2^) + (0.0485*P*)^2^ + 0.5445*P*] where *P* = (*F*_o_^2^ + 2*F*_c_^2^)/3
*S* = 1.03	(Δ/σ)_max_ = 0.001
6047 reflections	Δρ_max_ = 0.39 e Å^−^^3^
239 parameters	Δρ_min_ = −0.25 e Å^−^^3^
48 restraints	

### Special details

**Table:**

Geometry. All esds (except the esd in the dihedral angle between two l.s. planes) are estimated using the full covariance matrix. The cell esds are taken into account individually in the estimation of esds in distances, angles and torsion angles; correlations between esds in cell parameters are only used when they are defined by crystal symmetry. An approximate (isotropic) treatment of cell esds is used for estimating esds involving l.s. planes.

### Fractional atomic coordinates and isotropic or equivalent isotropic displacement parameters (Å^2^)

**Table:**

	*x*	*y*	*z*	*U*_iso_*/*U*_eq_	Occ. (<1)
S1	0.43796 (4)	0.38720 (2)	0.29828 (2)	0.02980 (8)	
O1	0.24466 (11)	0.32510 (7)	0.59768 (6)	0.0331 (2)	
N1	0.38490 (11)	0.20786 (7)	0.37310 (7)	0.02681 (19)	
C1	0.36366 (13)	0.30555 (8)	0.37431 (8)	0.0252 (2)	
C2	0.28925 (12)	0.35256 (8)	0.44102 (7)	0.0242 (2)	
C3	0.29554 (12)	0.45436 (8)	0.43292 (8)	0.0256 (2)	
C4	0.2295 (15)	0.5272 (10)	0.4954 (9)	0.0321 (5)	0.659 (2)
H4A	0.2554	0.5088	0.5612	0.038*	0.659 (2)
H4B	0.1169	0.5270	0.4838	0.038*	0.659 (2)
C4A	0.232 (3)	0.523 (2)	0.4994 (18)	0.0321 (5)	0.341 (2)
H4AA	0.3050	0.5316	0.5550	0.038*	0.341 (2)
H4AB	0.1347	0.4984	0.5198	0.038*	0.341 (2)
C5	0.2890 (2)	0.62862 (13)	0.47989 (14)	0.0319 (3)	0.659 (2)
H5A	0.2274	0.6752	0.5123	0.038*	0.659 (2)
H5B	0.3963	0.6337	0.5066	0.038*	0.659 (2)
C5A	0.2063 (4)	0.6169 (3)	0.4479 (3)	0.0319 (3)	0.341 (2)
H5AA	0.1771	0.6663	0.4921	0.038*	0.341 (2)
H5AB	0.1208	0.6095	0.3991	0.038*	0.341 (2)
C6	0.2800 (3)	0.65215 (15)	0.37652 (16)	0.0358 (4)	0.659 (2)
H6A	0.1733	0.6457	0.3492	0.043*	0.659 (2)
H6B	0.3129	0.7189	0.3678	0.043*	0.659 (2)
C6A	0.3481 (6)	0.6498 (3)	0.4029 (3)	0.0358 (4)	0.341 (2)
H6AA	0.4342	0.6544	0.4516	0.043*	0.341 (2)
H6AB	0.3291	0.7145	0.3768	0.043*	0.341 (2)
C7	0.3826 (19)	0.5846 (14)	0.3276 (11)	0.0324 (10)	0.659 (2)
H7A	0.3563	0.5888	0.2595	0.039*	0.659 (2)
H7B	0.4901	0.6053	0.3404	0.039*	0.659 (2)
C7A	0.396 (4)	0.583 (3)	0.324 (2)	0.0324 (10)	0.341 (2)
H7AA	0.3326	0.5955	0.2649	0.039*	0.341 (2)
H7AB	0.5045	0.5921	0.3135	0.039*	0.341 (2)
C8	0.36852 (13)	0.48275 (8)	0.35769 (8)	0.0270 (2)	
C9	0.21326 (13)	0.30278 (8)	0.51617 (8)	0.0249 (2)	
C10	0.09322 (12)	0.23048 (8)	0.49013 (7)	0.0247 (2)	
C11	0.04152 (14)	0.17248 (10)	0.55985 (8)	0.0323 (3)	
H11	0.0872	0.1772	0.6218	0.039*	
C12	−0.07579 (16)	0.10824 (11)	0.53899 (10)	0.0393 (3)	
H12	−0.1096	0.0685	0.5865	0.047*	
C13	−0.14437 (15)	0.10158 (11)	0.44890 (10)	0.0389 (3)	
H13	−0.2253	0.0576	0.4348	0.047*	
C14	−0.09461 (14)	0.15925 (11)	0.37946 (9)	0.0354 (3)	
H14	−0.1421	0.1551	0.3179	0.042*	
C15	0.02434 (13)	0.22300 (9)	0.39966 (8)	0.0297 (2)	
H15	0.0590	0.2617	0.3516	0.036*	
C16	0.46997 (13)	0.16863 (8)	0.31614 (8)	0.0266 (2)	
H16	0.5178	0.2077	0.2732	0.032*	
C17	0.49574 (13)	0.06571 (8)	0.31511 (8)	0.0249 (2)	
C18	0.58705 (14)	0.02651 (9)	0.25008 (8)	0.0295 (2)	
H18	0.6283	0.0665	0.2052	0.035*	
C19	0.61767 (16)	−0.07066 (10)	0.25078 (9)	0.0352 (3)	
H19	0.6790	−0.0971	0.2061	0.042*	
C20	0.55903 (16)	−0.12931 (9)	0.31650 (10)	0.0366 (3)	
H20	0.5809	−0.1957	0.3172	0.044*	
C21	0.46824 (17)	−0.09070 (10)	0.38127 (9)	0.0378 (3)	
H21	0.4284	−0.1309	0.4265	0.045*	
C22	0.43529 (15)	0.00601 (9)	0.38046 (8)	0.0318 (2)	
H22	0.3716	0.0317	0.4243	0.038*	

### Atomic displacement parameters (Å^2^)

**Table:**

	*U*^11^	*U*^22^	*U*^33^	*U*^12^	*U*^13^	*U*^23^
S1	0.03553 (16)	0.02847 (15)	0.02699 (14)	−0.00477 (11)	0.01203 (11)	−0.00210 (10)
O1	0.0397 (5)	0.0347 (5)	0.0252 (4)	−0.0062 (4)	0.0050 (3)	−0.0039 (3)
N1	0.0272 (4)	0.0253 (5)	0.0284 (4)	−0.0026 (4)	0.0051 (4)	−0.0034 (4)
C1	0.0254 (5)	0.0259 (5)	0.0248 (5)	−0.0038 (4)	0.0050 (4)	−0.0017 (4)
C2	0.0227 (5)	0.0255 (5)	0.0247 (5)	−0.0027 (4)	0.0048 (4)	−0.0016 (4)
C3	0.0234 (5)	0.0261 (5)	0.0276 (5)	−0.0023 (4)	0.0042 (4)	−0.0024 (4)
C4	0.0324 (7)	0.0280 (13)	0.0370 (12)	0.0004 (7)	0.0102 (7)	−0.0051 (9)
C4A	0.0324 (7)	0.0280 (13)	0.0370 (12)	0.0004 (7)	0.0102 (7)	−0.0051 (9)
C5	0.0309 (8)	0.0251 (7)	0.0393 (9)	0.0007 (7)	−0.0001 (6)	−0.0055 (6)
C5A	0.0309 (8)	0.0251 (7)	0.0393 (9)	0.0007 (7)	−0.0001 (6)	−0.0055 (6)
C6	0.0434 (13)	0.0235 (7)	0.0396 (11)	0.0008 (9)	−0.0026 (8)	−0.0002 (7)
C6A	0.0434 (13)	0.0235 (7)	0.0396 (11)	0.0008 (9)	−0.0026 (8)	−0.0002 (7)
C7	0.035 (3)	0.0269 (9)	0.0360 (14)	−0.0065 (16)	0.0068 (14)	0.0024 (8)
C7A	0.035 (3)	0.0269 (9)	0.0360 (14)	−0.0065 (16)	0.0068 (14)	0.0024 (8)
C8	0.0272 (5)	0.0261 (5)	0.0282 (5)	−0.0038 (4)	0.0047 (4)	−0.0013 (4)
C9	0.0247 (5)	0.0250 (5)	0.0258 (5)	−0.0002 (4)	0.0061 (4)	−0.0006 (4)
C10	0.0230 (5)	0.0275 (5)	0.0243 (5)	−0.0011 (4)	0.0057 (4)	−0.0007 (4)
C11	0.0327 (6)	0.0387 (7)	0.0260 (5)	−0.0081 (5)	0.0050 (4)	0.0032 (5)
C12	0.0385 (7)	0.0443 (8)	0.0359 (6)	−0.0144 (6)	0.0082 (5)	0.0059 (5)
C13	0.0325 (6)	0.0449 (8)	0.0396 (7)	−0.0137 (6)	0.0048 (5)	−0.0028 (6)
C14	0.0290 (6)	0.0480 (8)	0.0290 (6)	−0.0081 (5)	0.0018 (5)	−0.0038 (5)
C15	0.0270 (5)	0.0373 (6)	0.0252 (5)	−0.0033 (5)	0.0057 (4)	0.0012 (4)
C16	0.0276 (5)	0.0260 (5)	0.0266 (5)	−0.0026 (4)	0.0050 (4)	−0.0006 (4)
C17	0.0243 (5)	0.0256 (5)	0.0250 (5)	−0.0016 (4)	0.0027 (4)	−0.0024 (4)
C18	0.0316 (6)	0.0300 (6)	0.0277 (5)	−0.0004 (4)	0.0070 (4)	−0.0009 (4)
C19	0.0379 (6)	0.0329 (6)	0.0355 (6)	0.0056 (5)	0.0082 (5)	−0.0041 (5)
C20	0.0447 (7)	0.0261 (6)	0.0387 (7)	0.0029 (5)	0.0015 (6)	−0.0015 (5)
C21	0.0505 (8)	0.0284 (6)	0.0356 (6)	−0.0072 (5)	0.0101 (6)	0.0013 (5)
C22	0.0370 (6)	0.0292 (6)	0.0305 (5)	−0.0057 (5)	0.0108 (5)	−0.0036 (4)

### Geometric parameters (Å, º)

**Table:**

S1—C1	1.7485 (11)	C7—H7B	0.9900
S1—C8	1.7282 (12)	C7—C8	1.50 (2)
O1—C9	1.2241 (14)	C7A—H7AA	0.9900
N1—C1	1.3815 (15)	C7A—H7AB	0.9900
N1—C16	1.2804 (15)	C7A—C8	1.51 (4)
C1—C2	1.3758 (15)	C9—C10	1.4873 (16)
C2—C3	1.4323 (16)	C10—C11	1.3978 (16)
C2—C9	1.4930 (15)	C10—C15	1.3938 (15)
C3—C4	1.509 (16)	C11—H11	0.9500
C3—C4A	1.50 (3)	C11—C12	1.3821 (18)
C3—C8	1.3651 (16)	C12—H12	0.9500
C4—H4A	0.9900	C12—C13	1.3872 (19)
C4—H4B	0.9900	C13—H13	0.9500
C4—C5	1.537 (13)	C13—C14	1.3856 (19)
C4A—H4AA	0.9900	C14—H14	0.9500
C4A—H4AB	0.9900	C14—C15	1.3868 (17)
C4A—C5A	1.52 (3)	C15—H15	0.9500
C5—H5A	0.9900	C16—H16	0.9500
C5—H5B	0.9900	C16—C17	1.4597 (16)
C5—C6	1.521 (3)	C17—C18	1.3973 (15)
C5A—H5AA	0.9900	C17—C22	1.3978 (16)
C5A—H5AB	0.9900	C18—H18	0.9500
C5A—C6A	1.524 (6)	C18—C19	1.3874 (18)
C6—H6A	0.9900	C19—H19	0.9500
C6—H6B	0.9900	C19—C20	1.3860 (19)
C6—C7	1.521 (15)	C20—H20	0.9500
C6A—H6AA	0.9900	C20—C21	1.3883 (19)
C6A—H6AB	0.9900	C21—H21	0.9500
C6A—C7A	1.56 (3)	C21—C22	1.3852 (18)
C7—H7A	0.9900	C22—H22	0.9500
			
C8—S1—C1	91.70 (5)	C8—C7—H7B	109.0
C16—N1—C1	121.35 (10)	C6A—C7A—H7AA	110.7
N1—C1—S1	125.59 (8)	C6A—C7A—H7AB	110.7
C2—C1—S1	110.48 (8)	H7AA—C7A—H7AB	108.8
C2—C1—N1	123.77 (10)	C8—C7A—C6A	105 (2)
C1—C2—C3	113.31 (10)	C8—C7A—H7AA	110.7
C1—C2—C9	123.50 (10)	C8—C7A—H7AB	110.7
C3—C2—C9	123.16 (10)	C3—C8—S1	112.18 (9)
C2—C3—C4	127.2 (5)	C3—C8—C7	124.3 (6)
C2—C3—C4A	124.6 (9)	C3—C8—C7A	128.7 (11)
C8—C3—C2	112.26 (10)	C7—C8—S1	123.5 (6)
C8—C3—C4	120.5 (5)	C7A—C8—S1	119.2 (11)
C8—C3—C4A	123.2 (9)	O1—C9—C2	119.91 (10)
C3—C4—H4A	109.1	O1—C9—C10	120.87 (10)
C3—C4—H4B	109.1	C10—C9—C2	119.12 (9)
C3—C4—C5	112.7 (8)	C11—C10—C9	118.78 (10)
H4A—C4—H4B	107.8	C15—C10—C9	121.96 (10)
C5—C4—H4A	109.1	C15—C10—C11	119.14 (11)
C5—C4—H4B	109.1	C10—C11—H11	119.9
C3—C4A—H4AA	110.3	C12—C11—C10	120.29 (12)
C3—C4A—H4AB	110.3	C12—C11—H11	119.9
C3—C4A—C5A	106.9 (16)	C11—C12—H12	119.9
H4AA—C4A—H4AB	108.6	C11—C12—C13	120.25 (12)
C5A—C4A—H4AA	110.3	C13—C12—H12	119.9
C5A—C4A—H4AB	110.3	C12—C13—H13	120.1
C4—C5—H5A	109.5	C14—C13—C12	119.88 (12)
C4—C5—H5B	109.5	C14—C13—H13	120.1
H5A—C5—H5B	108.1	C13—C14—H14	119.9
C6—C5—C4	110.7 (5)	C13—C14—C15	120.16 (12)
C6—C5—H5A	109.5	C15—C14—H14	119.9
C6—C5—H5B	109.5	C10—C15—H15	119.9
C4A—C5A—H5AA	109.2	C14—C15—C10	120.28 (11)
C4A—C5A—H5AB	109.2	C14—C15—H15	119.9
C4A—C5A—C6A	112.1 (11)	N1—C16—H16	119.0
H5AA—C5A—H5AB	107.9	N1—C16—C17	122.01 (10)
C6A—C5A—H5AA	109.2	C17—C16—H16	119.0
C6A—C5A—H5AB	109.2	C18—C17—C16	119.57 (10)
C5—C6—H6A	109.7	C18—C17—C22	119.32 (11)
C5—C6—H6B	109.7	C22—C17—C16	121.06 (10)
H6A—C6—H6B	108.2	C17—C18—H18	119.9
C7—C6—C5	109.6 (7)	C19—C18—C17	120.19 (11)
C7—C6—H6A	109.7	C19—C18—H18	119.9
C7—C6—H6B	109.7	C18—C19—H19	119.9
C5A—C6A—H6AA	108.7	C20—C19—C18	120.23 (12)
C5A—C6A—H6AB	108.7	C20—C19—H19	119.9
C5A—C6A—C7A	114.0 (14)	C19—C20—H20	120.1
H6AA—C6A—H6AB	107.6	C19—C20—C21	119.79 (12)
C7A—C6A—H6AA	108.7	C21—C20—H20	120.1
C7A—C6A—H6AB	108.7	C20—C21—H21	119.8
C6—C7—H7A	109.0	C22—C21—C20	120.47 (12)
C6—C7—H7B	109.0	C22—C21—H21	119.8
H7A—C7—H7B	107.8	C17—C22—H22	120.0
C8—C7—C6	113.0 (11)	C21—C22—C17	119.98 (11)
C8—C7—H7A	109.0	C21—C22—H22	120.0
			
S1—C1—C2—C3	−1.73 (12)	C4A—C3—C8—C7A	−2 (2)
S1—C1—C2—C9	179.95 (9)	C4A—C5A—C6A—C7A	65.0 (19)
O1—C9—C10—C11	−14.66 (17)	C5—C6—C7—C8	44.1 (12)
O1—C9—C10—C15	161.26 (12)	C5A—C6A—C7A—C8	−39 (2)
N1—C1—C2—C3	173.90 (10)	C6—C7—C8—S1	165.8 (6)
N1—C1—C2—C9	−4.42 (18)	C6—C7—C8—C3	−11.9 (15)
N1—C16—C17—C18	178.62 (11)	C6—C7—C8—C7A	−164 (23)
N1—C16—C17—C22	−3.83 (18)	C6A—C7A—C8—S1	−169.1 (10)
C1—S1—C8—C3	1.38 (9)	C6A—C7A—C8—C3	10 (3)
C1—S1—C8—C7	−176.6 (8)	C6A—C7A—C8—C7	40 (21)
C1—S1—C8—C7A	−179.6 (15)	C8—S1—C1—N1	−175.30 (10)
C1—N1—C16—C17	178.88 (10)	C8—S1—C1—C2	0.23 (9)
C1—C2—C3—C4	−178.5 (6)	C8—C3—C4—C5	−14.7 (10)
C1—C2—C3—C4A	−176.3 (12)	C8—C3—C4A—C5A	22.6 (19)
C1—C2—C3—C8	2.81 (14)	C9—C2—C3—C4	−0.2 (6)
C1—C2—C9—O1	127.09 (13)	C9—C2—C3—C4A	2.0 (12)
C1—C2—C9—C10	−56.53 (15)	C9—C2—C3—C8	−178.86 (10)
C2—C3—C4—C5	166.7 (4)	C9—C10—C11—C12	176.41 (12)
C2—C3—C4A—C5A	−158.4 (7)	C9—C10—C15—C14	−175.46 (12)
C2—C3—C8—S1	−2.58 (13)	C10—C11—C12—C13	−0.7 (2)
C2—C3—C8—C7	175.4 (8)	C11—C10—C15—C14	0.45 (18)
C2—C3—C8—C7A	178.5 (17)	C11—C12—C13—C14	0.3 (2)
C2—C9—C10—C11	169.00 (11)	C12—C13—C14—C15	0.5 (2)
C2—C9—C10—C15	−15.07 (16)	C13—C14—C15—C10	−0.9 (2)
C3—C2—C9—O1	−51.06 (16)	C15—C10—C11—C12	0.37 (19)
C3—C2—C9—C10	125.31 (12)	C16—N1—C1—S1	3.22 (17)
C3—C4—C5—C6	47.5 (9)	C16—N1—C1—C2	−171.74 (11)
C3—C4A—C5A—C6A	−51.6 (16)	C16—C17—C18—C19	177.34 (12)
C4—C3—C4A—C5A	−11 (25)	C16—C17—C22—C21	−176.47 (12)
C4—C3—C8—S1	178.7 (6)	C17—C18—C19—C20	−0.6 (2)
C4—C3—C8—C7	−3.4 (10)	C18—C17—C22—C21	1.09 (18)
C4—C3—C8—C7A	−0.3 (18)	C18—C19—C20—C21	0.6 (2)
C4—C5—C6—C7	−62.7 (10)	C19—C20—C21—C22	0.3 (2)
C4A—C3—C4—C5	132 (26)	C20—C21—C22—C17	−1.1 (2)
C4A—C3—C8—S1	176.6 (11)	C22—C17—C18—C19	−0.25 (18)
C4A—C3—C8—C7	−5.5 (14)		

### Hydrogen-bond geometry (Å, º)

**Table:**

*D*—H···*A*	*D*—H	H···*A*	*D*···*A*	*D*—H···*A*
C18—H18···O1^i^	0.95	2.45	3.4034 (15)	176

Symmetry code: (i) *x*+1/2, −*y*+1/2, *z*−1/2.
